# Nursing Students’ Views of Nursing Education Quality: A Qualitative Study

**DOI:** 10.5539/gjhs.v7n2p351

**Published:** 2015-01-12

**Authors:** Fatihe Kermansaravi, Ali Navidian, Fariba Yaghoubinia

**Affiliations:** 1 Pregnancy Health Research Center, Zahedan University of Medical Sciences, Zahedan, Iran

**Keywords:** nursing education, nursing students, experience, content analysis

## Abstract

**Background:**

Nursing education is currently facing challenges related to the application of nursing knowledge in clinical environments and inability of students in application of nursing procedures in clinical settings. Nursing students themselves represent the best means of identifying these challenges. This study was conducted aimed to understand the nursing students’ viewpoints and experiences concerning the challenges and deficiencies of the nursing education system.

**Methods:**

This qualitative study that has been carried out adopting conventional qualitative content analysis approach, 40 senior nursing students with sufficient experience of educational situations participated through purposive sampling. Eight focus group discussions were done with volunteer nursing students from School of Nursing and Midwifery in Zahedan (Iran). All of the interviews and discussions were recorded and then analyzed using the conventional content analysis approach.

**Results:**

Three themes were emerged from data analysis including theoretical education, clinical skills, and the gap between theoretical education and clinical skills.

**Conclusions:**

The students’ views and experiences of nursing education quality (theoretical, clinical, and the gap between theoretical education and clinical skills) demonstrated a need to pay careful attention to the selection and recruitment of clinical teachers, and the assessment and control of their educational performance and clinical skills, as well as to determination of standards and validation of education quality.

## 1. Background

Universities are responsible for the disseminating and transferring of knowledge, as well as for providing specialized human resources. Also, they continually required to analyze and update their services, and to identify problems and challenges to allow them to optimize educational quality (Yazdankha-Fard, Pouladi, & Kamali, 2008).

The ongoing educational assessment of different scientific disciplines is critical, particularly for medical sciences. Educational assessment helps to identify constraints and obstacles, and to solve problems and identify strengths and weaknesses. The results of such assessments allow positive aspects to be upheld and effective solutions for potential problems to be implemented (Ziaee et al., 2006).

Many studies have indicated a need for the nursing education system to keep pace with continuous changes in nursing practice. The literature suggests that the results of apprenticeship are not satisfactory, which is indicated the need for more attention to this aspect (Adami & Kiger, 2005). Although new nursing graduates possess a strong theoretical background and knowledge, but they don’t have the necessary skills and dexterity which are required to solve problems that arise in clinical settings. Some studies have also indicated the existence of a vast gap between classical nursing and obstetric education and clinical care performance, suggesting that the present clinical education system does not provide students with the necessary clinical skills (Hadizadeh, Firoozi, & Shamaeyan-Razavi, 2005). The results of research on various clinical aspects indicate that students consider the quality of the education to be unsatisfactory. Reported deficits were including the instructors’ inabilities to apply theoretical principles in practical situations, inconsistency of apprenticeships in clinical wards, students being compelled to do other unrelated tasks, lack of proper evaluation by instructors, and a lack of congruence between theoretical learning and the clinical nursing services (Hadizadeh, Firoozi, & Shamaeyan-Razavi, 2005; Salehi et al., 2001; Hosani, Karimi, & Malekzade, 2005).

Studies in other countries have also identified a gap between theoretical learning and clinical nursing services is caused by clinical environment constraints, a lack of coordination between clinical settings and educational institutes, an obscure role for nursing instructors, defects in curriculum planning, and the clinical performance and role of the lecturers (Corlett, 2000; Ferguson, & Jinks, 1999).

Although the nursing students spend a long time in clinical settings, this cannot guarantee educational quality alone, because many variables influence the learning outcomes including the student’s personality, the clinical teachers and their skills, the ward personnel and their willingness to co- operate with the students, interpersonal relationships, attitudes, equipments, physical structure, and hierarchical relations, as well as other factors in the educational environment that are recognized in organizational and educational theories (Moattri & Remazani, 2009). The development and promotion of nursing education requires continuous monitoring for correcting weaknesses, and students as the outputs of this education are the best who identify these educational problems (Delaram, 2006; Hughe, Wad, & Peters, 1991).

Few studies have determined the students’ views of their educational problems and limitations; therefore the current study was done aimed to explain the experiences and views of nursing students about educational quality through the qualitative approach.

## 2. Methods

Current study is a qualitative research. To achieve the aim of the study and to explain the experiences and views of nursing students about educational quality, conventional content analysis approach was used. Participants included 40 bachelor fourth-year students nursing in School of Nursing and Midwifery (Zahedan) who were selected through purposive sampling in 2011. All of the students had adequate clinical experience of educational situations. Of the 50 students in fourth-year, 40 stated their willingness to participate in the research and were included in the study.

Data were collected through focus group discussions and 4 Goal-based guiding questionnaires. The questions were focused on the theoretical and clinical education. Based on the participants’ choice, discussions were conducted in classroom.

Eight focus group discussion sessions were done in line with the students’ curriculum. Before starting the sessions, the research aim, research nature, voluntary participation, privacy of information, and the anonymity of the participants were explained and guarantied, and all of the students agreed to their voices being recorded. Interviews with the students were carried out in four groups of 10 students, and each group had two 90–105 minute sessions. All the interviews were recorded and transcribed verbatim.

According to conventional content analysis approach, at first each focus group text was read again and again carefully in order to gain a universal and primary understanding of the important under-lined statements. Then, meaning units about participants’ experiences of nursing education, its problems and challenges existent in the interview text were determined. This procedure was repeated for all the focus group interviews. In the next phase, the meaning units were abstracted through condensation and were labeled as codes.

Participants’ statements and implicit concepts were used for coding. Codes were compared for similarities and differences, and then categorization of codes was done accordingly. Each theme was collated with the participants’ statements to produce an ultimate definition of the themes (Polit & Hungler, 1999; Holloway, & Wheeler, 2002).

In current study, credibility was established through prolonged engagement with participants, member checking, peer debriefing and review of data analysis with colleagues. For dependability, renewed coding of the text was carried out by colleagues who had experience in coding qualitative data. Moreover, the researchers documented research details in order to provide the possibility of external review. In addition, experts were invited to revise the themes to reach a consensus on related codes and the categorization of data. Focus group analysis is similar to other approaches, and the content analysis method was therefore used to analyze the qualitative data collected through the interviews (Salsaly, Parvizy, & Adib Haj Baghery, 2005; Braun, & Clark, 2006).

This research was carried out after getting ethical approval from research Ethics Committee, Zahedan University of Medical Sciences and with the permission and consent of the Dean of Nursing and Midwifery School.

## 3. Results

Most of the students were female (94 percent), with a mean age of 23.3±2.1 years old, and 95 percent of the students were single. After completing the interviews, 250 postulates were extracted and categorized into three main themes: theoretical education, clinical education, and the gap between clinical and theoretical education (Table 1).

**Table 1:** Themes, categories and subcategories

Meaning units	Condensed meaning units	Subcategories	Categories	Theme
Only the teachers speak in the class	centrality of teacher	The traditional teaching method	Teaching method	
We only write the handout in the class	Difficulty in learning process	Writing the handout		
We are not allowed to question in the class	One-way education and without feedback	Lack of participation of student		
The teaching aids were not used	Lack of utilization of teaching aids	Lack of utilization of teaching aids		
	
		Non applicability of some topics	Educational content (Teaching program)	**Theoretical education**
		Incomplete teaching of syllabus		
	
		Lack of mastery in course	Teacher’s characteristics	
		Disability in content transferring		
		Insufficient educational experience		
		Educational degree		

		The interval between taught content and apprenticeship	Defects of educational planning	**Clinical education**
		Repeat the apprenticeship		
		Lack of clinical education objectives		
		Mismatch of educational expectations with ward expectations		

		Insufficient of educational equipments and facilities	Defects of educational environment	
		Difficulty in doing of procedure due to large number of students		

		Professional Incompetency of clinical teacher	Clinical teacher
		evaluation criteria uncertainty	
		Lack of cooperation of clinical settings	

		Failure to implement the learned theory at the bedside	Application of theory in practice	**Theory-practice gap**
		Limitations in clinical practice due to diseases		
		Lack of Implementation of nursing process		
		Disability for implementation of nursing process		
		Mismatch of Learning content with clinical settings		

		Lack of application of scientific principles in patient care	Observing the scientific principles in practice	
		Routine performance in bedside		
		Lack of mastery and skills in clinical teacher		

Important topics in each text were identified and labeled as challenges in the nursing education.

**Theoretical education:** This theme was extracted from teaching methods, the curriculum, and the instructors. Regarding the teaching methods, one of the student commented: “the traditional teaching method, i.e., narration, was used in most classes”. Another student stated: “most of the instructors used one-way teaching methods; students were not permitted to participate in class discussions and most of the instructors believed that student participation was a waste of time”.

Concerning the note taking, one student commented:” the most instructors were merely concerned with finishing the class and preferred the students to take minutes, thus preventing opportunities to ask questions”. Another student stated: “he merely took notes to keep up with what the instructors taught them, without adding anything to their knowledge.

Regarding the curriculum, students stated “the subject titles were not completely covered and that what was taught had no applied value”. Many students experienced that the most of the theoretical credits were never applied, and the learning of them was a waste of time.

Concerning the instructors’ qualifications, students commented: “although the instructors’ knowledge background was satisfactory, but they didn’t have sufficient mastery of the subjects and their explanatory abilities were poor. Some students believed that instructors were selected purely on the basis of their degree, and an instructor with a Ph.D. would be preferred over one with an M.A., even if the Ph.D. instructor lacked teaching abilities and skills.

**Clinical education**: This theme was extracted from educational planning and environment, and clinical instructors. Most students believed that there was a lag between the taught topics and their apprenticeship, which rendered some of the apprenticeship redundant. They also noted that there was no match between the apprenticeship goals and the educational expectations on the one hand, and the wards’ expectations on the other hand.

One student commented: “I entered the surgery ward without passing any relevant theoretical credits, and we had consequently wandered about the ward, wasting time and missing out on opportunities for learning and gaining experience”. Another student stated: “instead of gaining practical skills and getting acquainted with the ward tasks, we had to become familiar with diseases and the nursing care related to these diseases, while some our classmate passed the same ward more than once”.

Regarding the agreement between educational and clinical expectations, one student commented: “we had performing nasogastric intubation when the ward nurse complained that they and the other students merely disturbed the ward routine”. Another student wished that the nurses and supervisors had been made aware of the students’ tasks so that, for instance, in addition to monitoring patients’ vital signs, they could also learn other clinical skills needed in every ward.

Regarding the educational environment, most students thought that the educational equipment and facilities were inadequate and that high student numbers and a lack of collaboration of medical system meant that there were no nursing procedures in place. Regarding the lack of procedures, one student commented: “the most of the procedures which were taught in classes could not be applied in the hospital environment”. Another factor criticized by the students was collaboration with the medical system. One student said that: “the hospital staff did not help us, and that while staff were able to share their experiences with the students, they were reluctant to guide the students and believed that the presence of students made situations worse”.

Another student said that: “the supervisors complained about the numbers of students in the wards, suggesting that the crowds caused disorder and disrupted patient treatment”. Students also emphasized the professional qualifications, skills, mastery and evaluation criteria of the clinical instructors. Most of the students thought that the clinical instructors were directly and indirectly an important factor in their learning.

One student suggested that the instructors’ knowledge was not up to date and that inexperienced instructors were selected for apprenticeship courses. Regarding the instructors’ skills, one student said that “we experienced difficulty in IV injection for one of the patients, but the skill and clinical dexterity of one of the female nurses improved their self-confidence and taught them to perform the procedure”.

The students indicated that: “the present assessment forms were not suitable for measuring students’ clinical skills during our apprenticeship, and did not cover all the practical skills in the various wards”. One student commented: “there was no logical relationship between the students’ apprenticeship scores and their ability levels”. Another student expressed: “the apprenticeship assessment lacked the standard practical assessment criteria, the assessment form was not specialized, that ward skills were not included in the forms, and that the students’ scores were thus incorrect”.

Based on the students’ experiences, the clinical environment also had some deficiencies, and according to one student, one of the main shortage related to the equipment and facilities needed to provide the patients with optimal services and care.

**The gap of Theoretical-clinical education:** This theme was emerged from the students’ emphasis on the integration of theoretical and clinical education, and from their views on the extent to which their theoretical education could be applied in real situations. The lack of coordination between theoretical and clinical education was a frequently reported problem in nursing education. All the students unanimously identified this problem as **a gap between theory and practice,** or **in** applying **nursing** processes in a clinical situation and applying scientific principles in a real clinical environment.

One student commented: “there were difficulties in applying the theoretical knowledge those we they had learned. The procedures carried out on patients did not match with what we had learned in theory, and we were guided by what the nurses did in the wards, rather than by what we had learned in the classes”. On applying the nursing process, one student stated: “I had to take note of the nursing processes in the wards, but I was unable to carry out the processes alone”.

In addition, some processes which were learned by nursing students were not used in hospitals. Students also mentioned the application of practical principles in caring for patients and the present ward routines. One student said that the unprofessional activities of ward nurses provided a poor educational example. For instance, nurses objected to students performing a technique correctly, and believed that performing procedures properly and according to the rules was only feasible in an educational environment, and that there was no need for such principles in real, practical situations, because there were many patients and few nurses.

One student commented: “what I had learned was a waste of time because procedures were carried out differently in real situations, and I consequently forgot the methods that I had been taught”.

## 4. Discussion

This was a qualitative study aimed to explaining the nursing students’ views and experiences concerning the problems and limitations of the current nursing education system. The students described the teaching methods as traditional and teacher-centered, inevitably resulting in minimal student participation. Although the goal of nursing education is to train skilled and experienced nurses who are capable of caring for patients in clinical environments (Peyrovi, Yadavar–Nikravesh, Oskouies, & Bertero, 2005), the present teaching and training methods do not equip students with decision-making skills.

Nursing education thus needs to focus more on teaching methods that encourage problem solving skills and develop critical thinking potential (Gonzalez et al., 2008). The nature of the nursing profession requires nurses to have a real understanding of the subjects and to be able to apply the theories in practical situations. Some studies indicated that active education methods significantly promoted deep-thinking skills, as well as perpetuating the learned subjects in students’ minds (Mirbagher Ajorpaz & Ranjbar, 2009; Carcich, & Rafti, 2007; Tiwari, & Chan, 2006).

Patel et al. showed that group discussions were more effective than lectures in terms of learning in clinical situations (Patel, Rocha, Branch, & Karlin, 2004). However, even though active learning methods have been shown to result in more effective and lasting learning, traditional teaching methods remain dominant in most educational institutes (Gonzalez et al., 2008). The prevalence of traditional methods is thought to derive from an easy-going culture, creating a security fringe for instructors and students, as well as reflecting a resistance to change. However, changes in educational methods and the promotion of educational quality need to be addressed.

Studies which were carried out about nursing education in Iran showed that the instructors and trainers play a pivotal role (Yamani, Yousfy & hangiz, 2006; Barazpor Zanjani, Fereidoonimogadam, & Loorizade, 2008). Teachers play a vital role in promoting any educational system, and the results of the present study identified poor teaching methods, insufficient knowledge, incorrect evaluation methods, and lack of suitable clinical backgrounds as obstacles and limitations.

These criticisms suggest that changes in educational strategies are required, in both theoretical and clinical situations (Gonzalez et al., 2008). Yamani et al. showed that using modern teaching methods, promoting the instructors’ scientific level, applying active educational solutions, and matching the theoretical education to professional needs could improve the nursing education (Yamani, Yousfy, & Hangiz, 2006). One study identified a lack of suitable educational equipment and facilities as a clinical education problem (Ouzouni, Nakakis, Koutsampasopoulos, & Kapadohos, 2010).

Students in the current study were also unsatisfied with the clinical education environment and equipment, and they assessed the equipment and facilities as mediocre (Sharif & Masaumi, 2005; Jouybari, Brahimi, & Sanagoo, 2006; Alavi & Bedi, 2006; Aeien, Alhani, & Anooshe, 2009). Learning in the clinical teaching environment is considered to be a fundamental aspect of the nursing education curriculum, enabling nursing students to integrate their theoretical education into clinical practice, and bridging the gap between theory and action in the nursing profession. The teaching environment must therefore be standardized to help nursing students to achieve this integration. Our results corroborate the findings of previous reports (Barazpor Zanjani, Fereidoonimogadam, & loorizade, 2008; Jouybari, Brahimi, & Sanagoo, 2006; Alavi & bedi, 2006; Aeien, Alhani, & Anooshe, 2009; Lambert, & Glacken, 2004).

Wotton and Gondo found that most students were not satisfied with the methods of evaluation, and students believed that clinical evaluations were not objective (Wotton & Gondo, 2004), suggesting the need to revise the processes and tools which are used for such evaluations. Most students believed that there was a lack of coordination between the educational and ward expectations. Some studies have indicated that setting realistic goals that fit the current facilities and conditions help to improve the quality of clinical education (Jouybari, Brahimi, & Sanagoo, 2006; Alavi & Bedi, 2006; Aeien, Alhani, & Anooshe, 2009).

Aeien et al. found that asking nursing students to perform activities requiring complex skills was unreasonable, and a close relationship between educational expectations and the expertise needed in clinical and practical situations was essential (Aeien, Alhani, & Anooshe, 2009). Students also identified problems with educational planning, such as students not being given clear job descriptions in some wards. This criticism was corroborated by Zighami et al., who found that 96 percent of students whom were asked believed that there was no clear and distinct job description in the wards (Zaighami, Faseleh Jahan-Miri, & Ghossbin, 2004). In contrast, a study of nursing students at Tehran University noted one of the strengths of the clinical education as student job descriptions being mentioned clearly in the set educational goals (Aeien, Alhani, & Anooshe, 2009). Experts believe that setting goals is an important and integral step in the learning process. Most students in the current study identified the professional qualifications of the instructors as a problem in terms of clinical education. Instructors’ performances are an important component of an effective clinical education, acting as a bridge between the theoretical and clinical aspects of nursing education.

Our findings are in agreement with those of other studies of instructors’ performances in Iran. In some such studies, half the students believed that the instructors’ performances were not satisfactory (Alavi & Abedi, 2006; Zaighami, Faseleh Jahan-Miri, & Ghossbin, 2004), while most students in another study assessed the instructors’ performances as good (Aeien, Alhani, & Anooshe, 2009). A study of instructors’ qualifications for nursing teaching found that students expected instructors to be responsible for the transfer of theoretical and practical skills, and should help nursing students to recognize their occupational role as a nurse (role imprinting) (YamaniN, Yousfy, & Hangiz, 2006; Karimi Mooneghi, Dabbaghi, & Vehvilainen-Julkunen, 2009). These studies therefore suggest that efficient and experienced instructors could help students to acquire the highest levels of clinical skills.

The existence of a gap between theory and action in nursing education and training is a long-standing problem, and the lag and discrepancy between the theoretical and clinical aspects of nursing education have caused much concern among instructors, professors, nurses and nursing students (Valizadeh, Abedi, Zamanzadeh, & Fathiazar, 2008). Most students in the current study pointed to the existence of a gap between theory and practice**,** a problem corroborated by other studies of this issue (Barazpor Zanjan, Fereidoonimogadam, & LoorizadeMR, 2008; Sharif, & Masaumi, 2005; Jouybari, Brahimi, & Sanagoo, 2006; Valizadeh, Abedi, Zamanzadeh, & Fathiazar, 2008).

One problem mentioned by the students in our study was that the nursing processes taught were not implemented in the wards. Salehy et al. found that most students believed that they lacked the necessary skills for caring for patients in accordance with the nursing process, and half of them said that they did not care for the patients based on the nursing process (Salehi et al., 2001). This suggests that the nursing education curriculum should be revised to fill the gap between the theoretical and practical aspects, and that the time lapse between the learned topics and apprenticeship should be shortened, giving students enough time to integrate the theoretical topics into their practical skills. Students’ views generally inferred that a high proportion of their time was allocated to memorizing large amounts of information that did not match the clinical requirements. Students also believed that active participation in the learning process was important, and claimed that they should learn the basic nursing knowledge to be able to solve the problems encountered in clinical situations.

## 5. Limitations of the Present Study

One limitation of the present study was that the data were not collected via individual detailed semi-structured interviews, and more information could have been acquired. Further studies are thus needed to overcome these limitations. It is also recommended that a questionnaire based on the findings of the current study could be used to confirm or complement these results in other nursing and obstetric colleges.

## 6. Conclusions

According to nursing students’ opinions and experiences, the factors influencing education quality have two dimensions. On the one hand, they emphasized the vital and determining role of hospital nursing staff in clinical education patterns, a lack of application of nursing processes, a failure to apply scientific principles, and incorrect education in clinical situations. On the other hand, students also recognized the existence of shortcomings in the curriculum, the fact that some theoretical subjects were not applied, and that traditional teaching methods continued to be used by instructors and trainers.

The findings of the present study suggest that the curriculum needs to be revised, and that scientific potential and abilities must be promoted, and standards and validation criteria should be determined for teaching hospitals. Most importantly, the students’ theoretical and practical knowledge should be integrated to upgrade the quality of the nursing education. Acknowledgments: Thanks to the Zahedan Medical Sciences University Deputy for Research Affairs and to all the students who participated in the study.
